# Elevated Blood Pressure in the Acute Phase of Stroke and the Role of Angiotensin Receptor Blockers

**DOI:** 10.1155/2013/941783

**Published:** 2013-01-31

**Authors:** Simona Lattanzi, Mauro Silvestrini, Leandro Provinciali

**Affiliations:** Neurological Clinic, Department of Experimental and Clinical Medicine, Marche Polytechnic University, Via Conca 71, 60020 Ancona, Italy

## Abstract

Raised blood pressure (BP) is common after stroke but its causes, effects, and management still remain uncertain. We performed a systematic review of randomized controlled trials that investigated the effects of the angiotensin receptor blockers (ARBs) administered in the acute phase (≤72 hours) of stroke on death and dependency. Trials were identified from searching three electronic databases (Medline, Cochrane Library and Web of Science Database). Three trials involving 3728 patients were included. Significant difference in BP values between treatment and placebo was found in two studies. No effect of the treatment was seen on dependency, death and vascular events at one, three or six months; the cumulative mortality and the number of vascular events at 12 months differed significantly in favour of treatment in one small trial which stopped prematurely. Evidence raises doubts over the hypothesis of a specific effect of ARBs on short- and medium-term outcomes of stroke. It is not possible to rule out that different drugs might have different effects. Further trials are desirable to clarify whether current findings are generalizable or there are subgroups of patients or different approaches to BP management for which a treatment benefit can be obtained.

## 1. Introduction

Raised blood pressure (BP) is common after acute stroke, whether of ischaemic or haemorrhagic type. It exists in more than three quarters of patients, of which about half have a history of hypertension [1], and it declines spontaneously in two-thirds of cases returning to prestroke levels over the first week. Its decrease usually occurs 4–10 days after stroke, but in a significant percentage of patients it falls by about 25–30% just within the first 24 hours; particularly when they are moved to a quiet room, they are allowed to rest and their bladder is empty [2].

Mechanisms and effects of elevated BP in this clinical setting have not been well understood. It might be attributable to either one more of the following conditions: preexisting, inadequately treated or undiagnosed hypertension, stress of hospitalization, raised intracranial pressure, haematoma expansion, damage to autonomic centers and abnormal baroceptor sensitivity, neuroendocrine response with activation of sympathetic nervous system, renin-angiotensin axis and/or glucocorticoid system, and myocardial changes [3–6].

Most of the studies, although not all, have found that high BP in the acute phase of stroke, whether measured as casual or 24 hours ambulatory readings, is associated with a poor outcome [7–9] and an increased risk of death and dependency [10–14]; a U-shaped relationship between BP values and outcome has been described in different studies [15–17]. Recent evidence suggests that not only BP but even its derived indices and other haemodynamic measures as mid blood pressure, mean arterial pressure, BP variability, heart rate, pulse pressure, and rate-pressure product are related to functional outcome [18–20]. The association is thought to be related to the early stroke recurrence and the development of cerebral edema and greater serious haemorrhagic transformation in ischaemic stroke [21, 22] and to the haematoma expansion in primary intracerebral haemorrhage [23].

While observational studies show that high BP is independently associated with a poor outcome, suggesting that it should be lowered, pathophysiology argues that lowering BP will reduce cerebral blood flow when cerebral autoregulatory mechanisms are impaired. Additionally, in acute ischaemic stroke the infarcted brain tissue may be surrounded by a “penumbra” zone of underperfused but viable tissue where cerebral blood flow extremely depends on the systemic BP and collaterals until the occluded artery is recanalized. Lowering BP carries the risk of jeopardizing the perfusion of this area leading to an increase of brain infarction or perihematoma ischaemia. Spontaneous thrombolysis may also occur, and the ischaemic area may become hyperaemic; at this stage a very high BP might cause propagation of infarct-related brain oedema or haemorrhagic transformation of the infarct. Unfortunately there is no sure clinical correlate of spontaneous thrombolysis, and in routine clinical care it is not possible to judge when it is better to leave a very high BP untreated or when it is necessary to intervene.

In summary, there is still debate in whether, when and how high BP should be lowered (epidemiological evidence) or not (pathophysiological concerns). Different antihypertensive drug classes might have differential effects [24] considering both their action in lowering BP and specific organ effects: for example *β*-blockers might be detrimental [25], and the use of calcium channel blockers was associated with a worsening of outcome in some studies, especially those testing intravenous formulation [26], perhaps because of a reduction in cerebral perfusion. 

Our purpose is to investigate through a research of the recent literature the effect of the angiotensin receptor blockers (ARBs) administered in the acute phase of stroke on death and dependency. 

## 2. Methods

### 2.1. Identification and Inclusion of Trials

We used Medline (1985 to December 2011; any language) to identify randomized controlled trials of ARBs in patients within 72 hours of stroke. We selected trials of more than 100 patients which assessed the effect on death or dependency and recorded coronary heart disease events or stroke (irrespective of whether BP lowering was considered the mechanism of action). Search terms were “blood pressure lowering”, “blood pressure reduction”, “antihypertensive”, or “hypertension” or “receptor, angiotensin/antagonists and inhibitors” or the name of all ARBs listed in the British National Formulary as keywords or text words. Limits were Medline publication type “clinical trial” or “controlled clinical trial” or “randomized controlled trial”, or “meta-analysis”. We also searched the Cochrane Collaboration and Web of Science databases and the citations in trials and meta-analysis. Randomized trials were included irrespective of participants' age, disease status, BP before treatment, and the use of other drugs. Confounded trials (in which ≥2 active treatment were compared in the absence of a control arm) were excluded.

### 2.2. Blood Pressure and Outcome

The authors extracted data independently from identified publications with respect to patients' number, sex, age, previous medical history, stroke subtype, time from stroke to enrollment, baseline BP, difference in BP between treatment and control groups, follow up period, grade of dependency, and death. Outcome events included stroke (all, fatal, and nonfatal), myocardial infarction (all), total vascular events (combined stroke, MI, and vascular death), and mortality (all-cause, vascular).

## 3. Results 

### 3.1. Trials

Three randomized placebo-controlled multicenter studies fulfilled the inclusion criteria (Table 1), each of which had been published [27–29]. The combined sample size was 3728 with almost two-thirds of the data coming from one study (SCAST). In two studies (ACCESS and PRoFESS) all patients recruited had ischaemic stroke, while in the SCAST trial 85.4% of patients had ischaemic and 13.5% haemorrhagic stroke; mean time from stroke to enrollment varied from 17.6 to 57.6 hours, and recruitment was limited to patients with high BP. Main characteristics of each trial are summarized in Table 2. 

In the ACCESS study, protocol considered a 7-day placebo controlled phase: treatment was started with 4 mg candesartan cilexetil daily or placebo on day 1; on day 2, dosage was increased to 8 or 16 mg candesartan cilexitil or placebo if BP exceeded 160 mmHg systolic or 100 mmHg diastolic; target was a 10–15% BP reduction within 24 hours. On day 7, a 24-hour BP profile was obtained in all patients: in those in the candesartan cilexitil group who showed a hypertensive profile (mean daytime BP > 135/85 mmHg) dosage was increased or an additional antihypertensive drug was added; in placebo arm, candesartan cilexitil was started in patients with hypertensive profile to lower BP to <140/90 mmHg (office BP) or < 135/85 mmHg (mean daytime BP), while those with a normotensive profile did not receive antihypertensive medication. Follow-up examinations were performed after 3, 6, and 12 months. 

The PRoFESS study compared the effect of telmisartan (80 mg daily) versus placebo and combined aspirin (25 mg twice daily) and extended release dipyridamole (200 mg daily) versus clopidogrel (75 mg daily) in a 2 × 2 factorial design in patients with recent ischaemic stroke followed up for a mean duration of 30 months. The subgroup analysis reviewed included only patients randomized within 72 hours of stroke onset, evaluated at 1 and 3 months.

In the SCAST trial, patients with diagnosis of stroke (ischaemic or haemorrhagic) presenting within 30 hours of symptom onset were allocated in a 1 : 1 ratio to treatment with candesartan or placebo. There was a fixed-dose escalation scheme: 4 mg on day 1, 8 mg on day 2 and 16 mg on days 3–7. Subsequent evaluations took place on day 7 and at 1 and 6 months; to avoid important differences in treatment during followup, candesartan was the advised antihypertensive agent and was provided free of charge.

In each trial there were no significant differences regarding the use of concomitant medication on hospital admission or during follow-up between the active treatment and placebo groups.

### 3.2. Baseline Findings

In the ACCESS trial mean BP on hospital admission was 198/103 and on study onset 189/99; baseline severity of stroke was not directly reported but patients had a mean Barthel Index (BI) of 62; no data are available about etiopathogenesis of strokes. In the PRoFESS subgroup analysis mean baseline BP was 147/84 mmHg and mean National Institutes of Health stroke scale (NIHSS) at admission was 3. According to TOAST classification, strokes were due to small artery occlusion in 59.5%, large-artery atherosclerosis in 20.9%, cardioembolism in 1.4%, and to undetermined or other determined etiologies in the remaining cases. In the SCAST study mean baseline BP was 171/90 mmHg and patients enrolled had a mean Scandinavian stroke scale (SSS) score of 41, equivalent to a NIHSS of 8 [30]. Strokes have been described following topographic rather than etiological criteria: according to Oxfordshire Community Stroke Project (OCSP) classification, strokes were distributed as total anterior syndrome in 8%, partial anterior syndrome in 48.7%, posterior syndrome in 14%, lacunar syndrome in 28.9%, unknown in <1%. Main baseline findings of the included studies are illustrated in Table 3.

### 3.3. Blood Pressure and Outcomes

In the ACCESS population no significant difference in BP was evident between the groups during the placebo-controlled phase and in the subsequent followup; at 7 days mean BP was 160/87 mmHg, at 3-months 150/85 mmHg and at 12 months 147/83 mmHg. Only two patients in the placebo arm had a normotensive profile on day 7 and did not receive the antihypertensive drug. In the PRoFESS subgroup analysis, telmisartan lowered significantly systolic BP (SBP) by 6 to 7 mmHg and diastolic BP (DBP) by 2 to 4 mmHg over the whole study course: on day 7, BP was135.3 (17.8)/78.4 (10.8) mmHg in treatment arm and 141.4 (17.0)/81.6 (11.0) mmHg in placebo group, with a difference of 6.1/3.2 mmHg (*P* < 0.0001); on day 30, mean BP was 135.7/79.6 mmHg versus 142.6/83.1 mmHg (treatment versus placebo) with a difference of 6.9/3.6 mmHg (*P* < 0.0001); on day 90, mean SBP was 134.5 (19.9) mmHg (treatment) versus 140.3 (19.0) (placebo) with a difference of 5.8 mmHg (*P* < 0.0001) and mean DBP was 79.2 (11.1) mmHg versus 81.5 (11.2) mmHg (treatment versus placebo) with a mean difference of 2.4 mmHg (*P* = 0.0002).

In the SCAST trial BP fell in both groups during treatment but was significantly lower in patients allocated candesartan than in those on placebo (*P* ≤ 0.001 for days 2–7); on day 7, mean BP was 147/82 mmHg (SD 23/14) in the candesartan group and 152/84 mmHg (SD 22/14) in the placebo group. The mean difference in SBP on day 7 was 5 mmHg (95% CI 3–7; *P* < 0.0001) and the mean difference in DBP was 2 mmHg (1–3; *P* = 0.001). During the 6-month followup, mean BP values were similar in the two groups, and at 6 months the mean BP was 143/81 mmHg in both groups.

In the ACCESS the BI revealed no significant difference on study onset and after 3 months (candesartan cilexetil versus placebo, day 0: 60.0 ± 30.2 versus 64.1 ± 27.5; 3 months: 87.0 ± 22.9 versus 88.9 ± 19.9). The cumulative 12-month mortality (candesartan cilexetil versus placebo: 2.9% versus 7.2%; *P* = 0.07) and the number of vascular events (candesartan cilexetil versus placebo: 9.8% versus 18.7%; *P* = 0.026) differed significantly in favor of the candesartan cilexetil group; the odds ratio was 0.475 (95% CI, 0.252 to 0.895). The clinical benefit was independent of BP values and mainly attributable to a lower incidence of myocardial ischaemic events. Drug tolerance and number or type of undesirable effects did not differ significantly between the groups.

In the PRoFESS subgroup analysis combined death or dependency (mRS at 30 days, with adjustment for baseline covariates) did not differ whether analyzed as an ordinal outcome (ordered mRS categories: 0, 1, 2, 3, 4–6 to maintain proportionality) (OR, 1.03; 95% CI, 0.84–1.26; *P* = 0.81) or with dichotomization of the data at the median (mRS 0-1 versus 2–6; OR, 1.00; 95% CI, 0.77–1.29). There was no significant difference between the treatment groups for the distribution of ordinal stroke events (fatal, dependent (mRS 2–5), independent (mRS 0,1), TIA, none), for the time to recurrence (*P* = 0.40) and, similarly, for other events (i.e., death, stroke recurrence, MI, and combined vascular events) at 7, 30, or 90 days. Serious adverse events were similar between telmisartan and control.

In the SCAST trial the analysis of the first co-primary effect variable, the cumulative risk of the composite endpoint of vascular death, stroke or myocardial infarction, showed no significant difference between candesartan and placebo (unadjusted analysis HR 1.09, 95% CI 0.84–1.41; *P* = 0.53; adjusted analysis HR 1.09, 0.84–1.41; *P* = 0.52; per-protocol analysis HR 1.11, 0.85–1.46; *P* = 0.46). Regarding the second co-primary effect variable, the functional outcome at 6 months, no significant difference was seen across the mRS categories (unadjusted ordinal regression analysis, OR 1.13, 95% CI 0.97–1.32; *P* = 0.12); similar results have been obtained in both the fixed dichotomy (mRS 3–6 versus 0–2) analysis (unfavourable outcomes in 35% of patients on candesartan and in 33% of patients allocated on placebo; OR 1.12, 0.90–1.41, *P* = 0.32; RR 1.06, 0.93–1.19, *P* = 0.39) and the sliding dichotomy analysis using the SSS scores at baseline (unfavourable outcomes in 56% of patients on candesartan and 52% of patients on placebo; OR 1.16, 95% CI 0.97–1.38, *P* = 0.11; RR 1.07, 95% CI 0.99–1.16, *P* = 0.11). For all the secondary outcomes assessed there were no significant differences between treatment and placebo: for all events (death from any cause, vascular death, ischaemic stroke, haemorrhagic stroke, all strokes, myocardial infarction, stroke progression, symptomatic hypotension, renal failure, and symptomatic venous thromboembolism: RR 1.47, 95% CI 1.01–2.13; *P* = 0.04), for SSS score at 7 days (*P* = 0.13) and for BI at 6 months (0.47). There were no significant differences between the groups for the adverse events reported by the investigators. 

Significant differences in BP values between treatment and placebo were therefore found in the PRoFESS and SCAST trials, but in only the first it remained significant during the follow-up; conversely, in the ACCESS study a significant difference was never found. 

In all trials no effect of the active treatment was seen on dependency, death and vascular events at one, three or six months; in the ACCESS study a decrease in cumulative mortality and number of vascular, mainly myocardial, events emerged in candesartan group at 12 months.

## 4. Discussion 

While the primary prevention of stroke through the treatment of hypertension is well established and evidence from randomized controlled trials suggests the use of antihypertensive agents for the secondary prevention of vascular events in patients with previous stroke or transient ischaemic attack, the management of BP immediately after stroke has been an enigmatic controversy for more than two decades, exemplified by a debate published back in 1985 [31], and still remains uncertain. In experimental studies on rats, candesartan has been shown to be neuroprotective with both reduction of neurovascular damage, demonstrated by decreased infarct size, hemoglobin content and oedema in the ischaemic brain hemisphere, and improvement of neurological outcome. These favorable effects were evident with an early post ischemic stroke drug administration, at doses that did not affect or moderately lowered BP [32, 33], suggesting a multimodal protective effect, partly due to BP lowering and partly due to pleiotropic, vascular and neuronal, actions. Homeostatic defense processes against postischaemic brain damage, or almost a part of them, are pressure dependent; presumably there are feedbacks and fine interrelationships among BP, the processes limiting the infarct size and the mechanisms involved in the recovery of ischemic tissue. On the other side, competitive inhibition of the binding of angiotensin (AT) II to AT_1_ receptors allows an unopposed activation of the AT_2_ receptors which has been hypothesized to be protective in focal cerebral ischaemia being responsible of several events as recruitment of cerebral collaterals, normalization of cerebrovascular autoregulation, enhancement of neuronal resistance to anoxia, inhibition of inducible nitric oxide synthetase, reduction of oxidative damage, prevention of apoptosis, promotion of angiogenesis and attenuation of inflammation, endothelial disfunction and prothrombosis [34, 35].

Translating these experimental findings to human stroke patients is quite difficult. The clinician who faces a patient in the acute phase of stroke with elevated BP has these main alternatives: he may choose to continue or not a preexisting antihypertensive drug or to introduce or not a new one. In the Continue Or Stop post-Stroke Antihypertensives Collaborative study [36], continuation compared with cessation of preexisting antihypertensive drugs for a two weeks period after acute stroke was not associated with a substantial reduction in two-week death or dependency, cardiovascular event rate or mortality at six months. Besides, clinical trials evaluating the effects of different BP lowering drugs administered in the acute phase of stroke have given conflicting results in respect of functional outcome, and the evidence to guide the practicing clinician is still rudimentary. Sharp reduction in arterial BP has been sought to determine a worse short and long-term prognosis [37, 38], while a moderate and caution reduction might be safe and even improve long term mortality and reduce recurrent vascular events [39, 40]. Not only the degree of BP reduction but even the nature of the pharmacological agent itself, considering its specific mechanism of action and in the perspective of possible drug-class related benefits, has been and still remain matter of debate. 

The purpose of this systematic review is to investigate the role of a specific class drug, the ARBs, early administered after stroke. None of the included studies demonstrated a significant benefit of active treatment on functional outcome and stroke recurrence at short and medium term, but some evaluations should be taken in account. Firstly, the mean BP at enrollment was much higher in the ACCESS study (189/99 mmHg) in respect to both the PRoFESS and SCAST trials (147/84 mmHg and 171/90 mmHg, resp.). Secondly, relevant differences existed in stroke severity: patients enrolled in the PRoFESS had very mild stroke in respect to those participating to the SCAST and above all to the ACCESS who had a significantly greater impairment. Thirdly, the effect of treatment on BP: candesartan did not alter BP in the ACCESS while in the PRoFESS, despite the fact that BP at baseline was reasonably well controlled, telmisartan further reduced it; also in the SCAST trial BP fell in both treatment and placebo but was significantly lower in patients allocated candesartan although difference disappeared during the followup. Finally, the achievement of endpoints: neither functional outcome nor number of vascular events have been positively affected by treatment but in the ACCESS study, although the primary outcome (disability at 3 months) was neutral, treatment with candesartan was associated with a significant reduction in secondary outcome including the 12-month mortality and vascular events. Taking into account the U-shaped relationship between BP and outcome in acute stroke, the main advantage deriving from lowering BP treatment would be expected in the ACCESS trial which included patients with severely elevated BP. Even in this study, however, 3-month outcome has not been influenced by treatment and the reduction in the number of vascular events observed in favor of the candesartan cilexitil group was mainly due to a lower incidence of myocardial ischaemic events but not of recurrent cerebral ischaemic events which did not significantly contribute to the difference in cardiovascular morbidity and mortality. Much more surprising is the fact that in this study BP has not been affected by treatment, raising the question around the existence of a drug specific effect of the AT_1_ receptor blockade, beyond the hemodynamic activity, able to modulate vascular remodeling and to affect cardiovascular survival with a benefit that did not arise immediately but appeared to increase over the time. Analogous data have been acquired from various cardiac intervention studies suggesting the hypothesis that early neurohumoral inhibition has similar beneficial effects in both cerebral and myocardial ischaemia, although the underlying mechanism is not resolved. These findings, however, have not been confirmed in the PRoFESS and SCAST trials, and such discrepancies may simply reflect a false positive finding of a small trial which was stopped prematurely on the basis of an interim analysis, requiring more investigations.

Taking their similarities and differences, the studies we have considered raise doubts over the hypothesis of a specific effect of angiotensin receptors blockade in acute stroke; moreover, conclusions are fully compatible with those of a recent meta-analysis [29] and a regularly update Cochrane survey [41] of randomized controlled trials of BP lowering drugs in acute stroke. According to currently available evidence there is no clear evidence of benefit for routine BP lowering treatment in the acute phase of stroke; many pitfalls, however, still remain to explain. Firstly, BP management in this clinical setting may have to be tailored with respect to the underlying etiology, and other parameters than baseline BP may need to taken into consideration [42, 43]. Patients with atherosclerotic or lacunar strokes are often affected by chronic hypertension with subsequent arterial stiffness and shift of the cerebral blood flow autoregulatory curve to the right. Consequently, they may tolerate elevated BP in acute stroke more efficiently, and BP values considered “normal” for the general population may be inadequate to perfuse the ischaemic brain. These patients usually present also diffuse atherosclerotic lesions in cerebral vessels which compromise the patency of collateral circulation: high BP may be needed to enhance perfusion of the ischaemic penumbra zone. On the contrary, in the cardioembolic strokes patients may only have atrial fibrillation in absence of arterial hypertension or significant atherosclerotic stenosis and may need only moderately elevated BP to promote perfusion through a patent collateral circulation; moreover, cardioembolic strokes tend to be of larger size with a higher risk of edema and hemorrhagic transformation in case of raised levels of BP. Unfortunately, the trials considered neither reported nor stratified outcome data according to the aetiopathogenesis of strokes. Secondly, the interrelationships between baseline BP, stroke severity, and administration of antihypertensive agents have not been well understood but an interaction could not be excluded; in the PRoFESS study the failure to show beneficial effect of telmisartan may reflect that patients had only mild hypertension and mild stroke. Thirdly, favourable effect of treatment may show a significant time interaction; a post hoc analysis of the main PRoFESS study indicated that recurrence was lower with telmisartan after the first six months of treatment [44], and in the ACCESS benefit on overall mortality and vascular events did not arise immediately but instead appeared to increase during follow up. The included studies presented followup ranging from 3 to 12 months and have not been designed to investigate long term effects of treatment. 

Although a benefit of early treatment has not emerged, it is noteworthy that a substantial safety has resulted without difference in number or type of undesirable effects in treatment and placebo groups; since chronic lowering BP reduces strokes recurrence [45, 46] it may be safe to start such treatment even acutely in selected subgroups. Many other issues like the timing of starting the treatment, the degree, and rapidity of BP reduction, also taking account of the initial level, and the formulation, route of administration, and doses of different pharmacological agents should be further addressed; it is not possible to rule out that different drugs might have different effects. 

Our analyses are not ideal in some respects. Firstly, trial-level data rather than individual patients' data were assessed since the latter were not available to us; analyses based on individual patients' data are generally superior and allow subgroup analyses to be performed. Secondly, the inhomogeneity of the patients' population and of the design of the study methods regarding cut offs for BP, stroke severity, time of assessment of the outcome variables; for the PRoFESS study, we have considered a subgroup of the patients entered into the main large secondary prevention trial, such that patients' characteristics reflect the inclusion criteria for a study of vascular prophylaxis rather than acute intervention. Thirdly, we could not assess the effect of lowering BP in patients with different subtypes of strokes since trials did not report these data separately. 

## 5. Conclusion and Future Research

The currently available studies did not identify any clear indication that treatment with the ARBs is beneficial in patients with acute stroke and raised BP on functional outcome and stroke recurrence at short and medium term. Many issues, however, should be still considered; two large studies involving more than 2500 patients with acute ischaemic (ENOS) and haemorrhagic stroke (ENOS, INTERACT 2) [47, 48] are ongoing, and it is favorable these and future trials will help to clarify many unresolved questions and whether there are subgroups of patients or different approaches to BP management for which a treatment benefit can be obtained. 

## Figures and Tables

**Table 1:** Included trials.

Study	Year	Study design	Antihypertensive agent
Acute Candesartan Cilexetil Therapy in Stroke Survivors (ACCESS) [27]	2003	Prospective, double-blind, placebo-controlled, randomized, multicenter phase II study.	Candesartan cilexitil
Prevention Regimen for Effectively Avoiding Second Strokes (PRoFESS) [28]*	2008	Prospective, double-blind, placebo-controlled, randomized, multicenter phase III study.	Telmisartan
The angiotensin-receptor blocker candesartan for treatment of acute stroke (SCAST) [29]	2011	Prospective, double-blind, placebo-controlled, randomized, multicenter phase III study.	Candesartan cilexitil

*In the PRoFESS trial a subgroup analysis has been considered.

**Table 2:** Main characteristics of the included trials.

Study	ACCESS [27]	PRoFESS [28]*	SCATS [29]
Main inclusion criteria	Motor deficit, cerebral CT scan excluding intracranial hemorrhage, onset of symptoms within 72 hours, necessity to treat hypertension according to current recommendations^#^.	Ischemic stroke within 72 hours from onset of symptoms, age older than 55 years or age 50–54 years if 2 additional vascular risk factors present, seated systolic BP 121 to 180 mmHg, seated diastolic BP ≥110 mmHg, neurological and clinical stability.	Patients aged 18 years or older with a clinical diagnosis of stroke (ischaemic or haemorrhagic), presenting within 30 hours of symptom onset and with systolic BP higher than 140 mmHg.

Relevant exclusion criteria	Age ≥85 years, occlusion or ≥70% stenosis of the internal carotid artery, malignant hypertension, manifest cardiac failure (NYHA class III and IV), high-grade aortic or mitral stenosis, unstable angina pectoris, contraindications against candesartan cilexetil.	Dysphagia preventing oral medication, mRS >3 at time of randomization, severe known renal insufficiency or renal artery stenosis or coronary artery disease or recent MI, hyperkalemia, uncorrected volume or sodium depletion, schedule for carotid endarterectomy, currently using or needing ARB	SSS consciousness score ≤2, premorbid mRS ≥4, clear indication for or contraindications to or current treatment with an ARB.

Treatment design	Candesartan cilexetil (4–16 mg daily according to BP levels)^+^.	Telmisartan (80 mg daily).	Candesartan cilexetil (4 mg on day 1, 8 mg on day 2 and 16 mg on days 3–7)^†^.

Follow-up evaluation	On day 7, at 3, 6, and 12 months.	On day 7, at 1, and 3 months.	On day 7, at 1, and 6 months.

Primary endpoints	Case fatality and disability (measured as BI) at 3 months.	Combined death or dependency (measured as mRS) at 30 days.	Composite endpoint of vascular death, nonfatal MI or nonfatal stroke and functional status (measured as mRS) at 6 months.

Secondary endpoints	Overall mortality and cerebrovascular and cardiovascular events at 12 months.	Overall mortality and cerebrovascular and cardiovascular events at 7, 30, and 90 days.	Stroke progression^‡^; neurological status at 7 days (measured as SSS); overall mortality, cerebrovascular and cardiovascular events, functional outcome (measured as BI) at 6 months.

*In the PRoFESS trial a subgroup analysis has been considered.

^#^This was assumed when the mean of at least 2 blood pressure measurements was ≥200 mmHg systolic and/or ≥110 mmHg diastolic 6 to 24 hours after admission or ≥180 mmHg systolic and/or ≥105 mmHg diastolic 24 to 36 hours after admission.

^+^Candesartan cilexetil 4 mg daily on day 1; on day 2, dosage was increased to 8 or 16 mg if blood pressure exceeded 160 mmHg systolic or 100 mmHg diastolic. At the end of the placebo-controlled 7-days phase, in candesartan cilexetil-treated patients the dosage was increased or an additional antihypertensive drug was added only in the case of a hypertensive profile (mean daytime blood pressure ≥135/85 mm Hg). In placebo-treated patients candesartan cilexetil was started only in presence of a hypertensive profile.

^†^Dose adjustments were made if systolic blood pressure was lower than 120 mmHg or when clinically indicated.

^‡^Stroke progression was defined as a neurological deterioration of 2 or more points on the SSS occurring within the first 72 h of stroke onset and believed to be caused by the index stroke, after exclusion of recurrent stroke or systemic reasons for deterioration.

ARB: angiotensin receptor blocker; BI: barthel index; BP: blood pressure; MI: myocardial infarction; mRS: modified ranking scale; NYHA: New York Heart Association; SSS: Scandinavian stroke scale.

**Table 3:** Patients characteristics at enrollment.

Study	ACCESS [27]	PRoFESS [28]*	SCATS [29]
	treatment/placebo	treatment/placebo	treatment/placebo
Number of patients	173/166	647/713	1017/1012
Age	68.3 (9.3)/67.8 (9.4)	66.8 (8.8)/67.1 (9.2)	70.8 (11.2)/71.0 (11.0)
Male (%)	86 (49.7)/86 (51.8)	420 (64.9)/464 (65.1)	612 (60.2)/564 (55.7)
Clinical history			
Previous stroke/TIA	NA	160 (24.7)/184 (25.8)	252 (24.8)/204 (20.2)
Atrial fibrillation	NA	10 (1.6)/14 (2.0)	190 (18.7)/186 (18.4)
Hypertension	NA	453 (70.0)/503 (70.6)	676 (66.5)/670 (66.2)
Diabetes mellitus	67 (38.7)/58 (35.0)	176 (27.2)/198 (27.8)	163 (16.0)/157 (15.5)
Hyperlipidemia	74 (42.8)/75 (45.2)	264 (40.8)/283 (39.7)	NA
Ischemic heart disease	38 (22)/32 (19.3)	95 (14.7)/104 (14.6)	NA
Time from stroke (hours)	29.9/29.7	57.6 (16.8)/57.6 (16.8)	17.6 (8.1)/17.9 (8.1)
Blood pressure (mmHg)			
Systolic	188 (20.9)/190 (19.7)	146 (16.2)/147 (16.3)	171.2 (19.0)/171.6 (19.2)
Diastolic	99 (14.9)/99 (13.0)	84 (10.1)/84 (10.2)	90.3 (13.9)/90.6 (14.2)
Clinical severity	60.0 (30.2)/64.1 (27.5) at BI	2.9 (2.8)/3.1 (2.9) at NIHSS	40.6 (12.3)/40.5 (12.6) at SSS

*In the PRoFESS trial a subgroup analysis has been considered.

Data are *n* (%) or mean (SD). NA: not assessed. BI: Barthel Index; mRS: modified ranking scale; NIHSS: National Institutes of Health Stroke Scale; SSS: Scandinavian stroke scale.
